# Validation of the Fitbit One^®^ for physical activity measurement at an upper torso attachment site

**DOI:** 10.1186/s13104-016-2020-8

**Published:** 2016-04-12

**Authors:** Keith M. Diaz, David J. Krupka, Melinda J. Chang, Jonathan A. Shaffer, Yao Ma, Jeff Goldsmith, Joseph E. Schwartz, Karina W. Davidson

**Affiliations:** Center for Behavioral Cardiovascular Health, Department of Medicine, Columbia University Medical Center, 622 West 168th Street, PH 9-319, New York, NY 10032 USA; Department of Psychology, University of Colorado Denver, Denver, CO USA; Department of Biostatistics, Mailman School of Public Health, Columbia University, New York, NY USA; Department of Psychiatry, Stony Brook University, New York, NY USA

**Keywords:** Physical activity, Validation studies, Accelerometer, Walking, Fitbit, Energy expenditure

## Abstract

**Background:**

The upper torso is recommended as an attachment site for the Fitbit One^®^, one of the most common wireless physical activity trackers in the consumer market, and could represent a viable alternative to wrist- and hip-attachment sites. The objective of this study was to provide evidence concerning the validity of the Fitbit One^®^ attached to the upper torso for measuring step counts and energy expenditure among female adults.

**Results:**

Thirteen female adults completed a four-phase treadmill exercise protocol (1.9, 3.0, 4.0, and 5.2 mph). Participants were fitted with three Fitbit^®^ trackers (two Fitbit One^®^ trackers: one on the upper torso, one on the hip; and a wrist-based Fitbit Flex^®^). Steps were assessed by manual counting of a video recording. Energy expenditure was measured by gas exchange indirect calorimetry. Concordance correlation coefficients of Fitbit-estimated step counts to observed step counts for the upper torso-attached Fitbit One^®^, hip-attached Fitbit One^®^ and wrist-attached Fitbit Flex^®^ were 0.98 (95 % CI 0.97–0.99), 0.99 (95 % CI 0.99–0.99), and 0.75 (95 % CI 0.70–0.79), respectively. The percent error for step count estimates from the upper torso attachment site was ≤3 % for all walking and running speeds. Upper torso step count estimates showed similar accuracy relative to hip attachment of the Fitbit One^®^ and were more accurate than the wrist-based Fitbit Flex^®^. Similar results were obtained for energy expenditure estimates. Energy expenditure estimates for the upper torso attachment site yielded relative percent errors that ranged from 9 to 19 % and were more accurate than the wrist-based Fitbit Flex^®^, but less accurate than hip attachment of the Fitbit One^®^.

**Conclusions:**

Our study shows that physical activity measures obtained from the upper torso attachment site of the Fitbit One^®^ are accurate across different walking and running speeds in female adults. The upper torso attachment site of the Fitbit One^®^ outperformed the wrist-based Fitbit Flex^®^ and yielded similar step count estimates to hip-attachment. These data support the upper torso as an alternative attachment site for the Fitbit One^®^.

## Findings

### Background

In recent years, wearable devices containing accelerometers, such as those made by Fitbit^®^ (e.g. Fitbit One^®^, Fitbit Flex^®^), have been widely introduced into the consumer market as physical activity trackers. These trackers can interface wirelessly with mobile phones and manufacturer-established websites to allow consumers to monitor and track their physical activity in real-time. The relatively low cost, interface capabilities, ease of use, and wide commercial availability of these activity trackers may ultimately change the way researchers and clinicians alike monitor their patients’ physical activity by providing remote access to patient-generated data.

Consumer-grade wireless physical activity trackers including those made by Fitbit^®^ offer models designed for attachment at either the wrist or hip. A 2015 systematic review by Evenson et al. [1]. identified 22 studies that have assessed the accuracy of consumer-grade activity trackers (e.g. Fitbit^®^, Jawbone^®^) of which two concurrently examined the accuracy of both the hip-based and wrist-based models made by Fitbit^®^ [2, 3]. In a 1500-step trial of treadmill walking at 3.0 mph among apparently healthy adults, step counts from the hip-based model (Fitbit One^®^; mean ± SD 1497.0 ± 10.7 steps; 0.2 ± 0.7 % error) were more accurate than step counts from the wrist-based model (Fitbit Flex^®^; 1378.0 ± 142.7 steps; 8.9 ± 9.5 % error) [2]. More recently, in a laboratory-based protocol of treadmill walking and running among apparently healthy adults, we reported that the Fitbit One^®^ outperformed the Fitbit Flex^®^ with relative percent errors of 0.6  versus 9.3 % for step counts and 6.0 versus 18.0 % for energy expenditure, respectively [3]. Informed in part by these findings, Evenson et al. concluded in their evaluation of the literature that hip-based activity trackers perform better than activity trackers worn on the wrist [1].

The hip has been the conventional attachment site for accelerometers because of its proximity to the human body’s center of mass (hence providing a more accurate measure of activity), nevertheless participant compliance with hip-based accelerometer wear has been recognized as a major issue due to the reported discomfort and/or inconvenience of wearing a device on the hip over time [4, 5]. In an effort to improve participant compliance to accelerometer wear over extended periods, population-based studies including the National Health and Nutrition Examination Survey (NHANES), Dallas Heart Study, and the UK Biobank project have recently moved the accelerometer to a wrist-worn attachment site [5–7]. However, in light of the lower accuracy of the wrist attachment site which some have attributed to smaller amounts of upper body motion during locomotor activities compared to the lower body [8], identification of alternative attachment sites that could improve participant compliance relative to hip-based wear without compromising device accuracy may be warranted. The upper torso is recommended by the Fitbit^®^ manufacturer as an attachment site for females (bra attachment) and could represent a viable alternative to wrist and hip attachment sites that may be conducive to better compliance than hip-based monitoring while providing more accurate estimates of physical activity than wrist-based monitoring. To our knowledge, no study has evaluated the accuracy of the Fitbit One^®^ attached to the upper torso. The purpose of this study, therefore, was to provide validity evidence supporting the use of the Fitbit One^®^ attached to the upper torso for measuring step counts and energy expenditure during treadmill walking and running among apparently health women. We hypothesized that the upper torso attachment site of the Fitbit One^®^ would: (1) yield a relative error of less than 3 % for steps counts (but not energy expenditure) across all walking/running speeds, an accepted criterion for laboratory-based validation studies [9–11], (2) yield comparable step count and energy expenditure estimates to the hip attachment site of the Fitbit One^®^ and (3) outperform the wrist-based Fitbit Flex^®^.

### Methods

#### Study population

We recruited a convenience sample of 24 apparently healthy adult participants that included staff and students at Columbia University Medical Center and former participants in research studies conducted at Columbia University Medical Center. Participants were enrolled between October 2013 and February 2014. A variety of recruitment tools were used including personal contact, flyers, phone, and email. Participants qualified for this study if they were ≥18 years of age and able to walk without human assistance or walking aids. Participants with a mobility-limiting health condition, a history of any chronic medical condition (including cardiovascular disease, hypertension, diabetes, and hypercholesterolemia), or ≥2 cardiovascular risk factors [middle- or older-aged (>45 years for males; >55 years for females), postmenopausal, family history of heart disease, current smoker, physically inactive (<3 days/week of moderate-vigorous physical activity for >30 min), and a self-reported history of being overweight] were excluded. For the current analysis, we excluded male participants in whom upper torso attachment has not been recommended by the manufacturer (n = 11), leaving a final sample size of 13 female participants. The study adhered to the guidelines set forth by the Declaration of Helsinki and was approved by the institutional review board of Columbia University Medical Center. All participants provided informed consent.

#### Study protocol

Participants completed a four-phase treadmill exercise protocol under laboratory conditions after having fasted for 6 h and refrained from exercise, alcohol, and use of any stimulants (caffeine, tobacco, and medication) in the previous 24 h. The protocol consisted of walking at slow (1.9 mph), moderate (3.0 mph), and brisk (4.0 mph) paces; and light running (5.2 mph). Each phase was 6 min in duration with a 3-min rest period between each stage. Participants were fitted with three Fitbit^®^ activity trackers: (1) a Fitbit One^®^ on the upper torso via attachment to the center of their bra using the manufacturer-provided silicone clip; (2) a Fitbit One^®^ on the right hip, positioned over the right anterior iliac spine via attachment to an elastic belt using the manufacturer-provided silicone clip; and (3) a Fitbit Flex^®^ fitted to the right wrist using the manufacturer provided wristband and positioned on the dorsal aspect of the wrist, just proximal to the radial and ulnar processes. The Fitbit One^®^ and Fitbit Flex^®^ trackers are microelectromechanical triaxial accelerometers with identical mechanical features that convert accelerations to step counts and energy expenditure using proprietary algorithms.

Steps were assessed by manual counting of a video recording. A 720p resolution at 60 frames per second video camera (Flip MinoHD M3160 camcorder; Cisco Systems, San Jose, California) was positioned approximately two feet to the back left corner of the treadmill so that observed steps were counted from a lateral view. A digital clock was displayed in front of the camera to give real time. Energy expenditure was assessed by gas exchange indirect calorimetry (Ultima CPX, MedGraphics, St. Paul, Minnesota) using a nose-clip and mouthpiece attached to a pneumotach/gas-sampling port.

#### Equipment synchronization

For data collection, one desktop computer was used to initialize and download all Fitbit^®^ trackers. The Fitbit^®^ trackers adopted the time of the computer and the internal times of the metabolic system and digital clock were also synchronized with this computer. All devices and equipment were synchronized approximately 10 min before the start of each session.

#### Data processing

Data from the Fitbit^®^ trackers were downloaded to the web-based manufacturer application. Minute-by-minute estimated step counts and energy expenditure data were then extracted using Fitabase (Small Steps Labs, San Diego, California). Observed steps for each 1-min epoch were counted from the video recording using a manual tally counter by two observers (Cronbach’s alpha = 0.999) and were averaged. Recordings were viewed using Windows Media Player version 10 (Microsoft Corporation, Redmond, Washington) and steps were measured to the nearest heel strike. Breath-by-breath measures of energy expenditure from gas exchange indirect calorimetry were aggregated to 1-min epochs.

#### Statistical analyses

Estimated step counts and energy expenditure from the Fitbit^®^ trackers were compared to the criterion measures of observed step counts and energy expenditure from indirect calorimetry, respectively. All minutes (minutes 1–6) from each stage were included in analyses, totaling 306 person-minutes of observation (6 person-minutes were not collected during the running phase for one participant due to indirect calorimetry equipment failure). Relative percent errors across each phase (slow, medium, and fast walking phases; running phase) were calculated relative to the criterion measures. A mean relative percent error of less than 3.0 % was selected as the criterion validity threshold in accordance with previous laboratory-based validation studies [9–11]. The level of agreement of Fitbit-estimated step counts and energy expenditure relative to the criterion measures were further assessed by calculating Lin’s concordance correlation coefficient (phases aggregated) [12]. Bland and Altman plots were also constructed to visually assess agreement of Fitbit^®^ tracker estimates with the criterion measures [13]. Finally, paired sample t tests were used to evaluate speed-specific mean differences in step counts and energy expenditure between the upper torso and (1) the criterion measures, (2) the hip attachment site of the Fitbit One^®^, and (3) the wrist-based Fitbit Flex^®^. In an effort to control for multiple testing associated with the large number of hypothesis tests conducted, we set the level of significance to 0.001. Data analyses were conducted using SAS version 9.3 (SAS Institute, Cary, NC, USA) and SPSS version 23 (SPSS Inc, Chicago, IL).

### Results

#### Participant characteristics

Among the 13 participants who completed the study, the mean age was 32.0 ± 9.2 years (range 20–54 years), 6 (46.2 %) self-identified their race as white, and 7 (53.8 %) were of Hispanic ethnicity. Participants’ body mass index (BMI) ranged from 19.6 to 29.9 kg/m^2^, with a mean of 24.2 ± 3.4 kg/m^2^.

#### Step counts

**Fig. 1:**
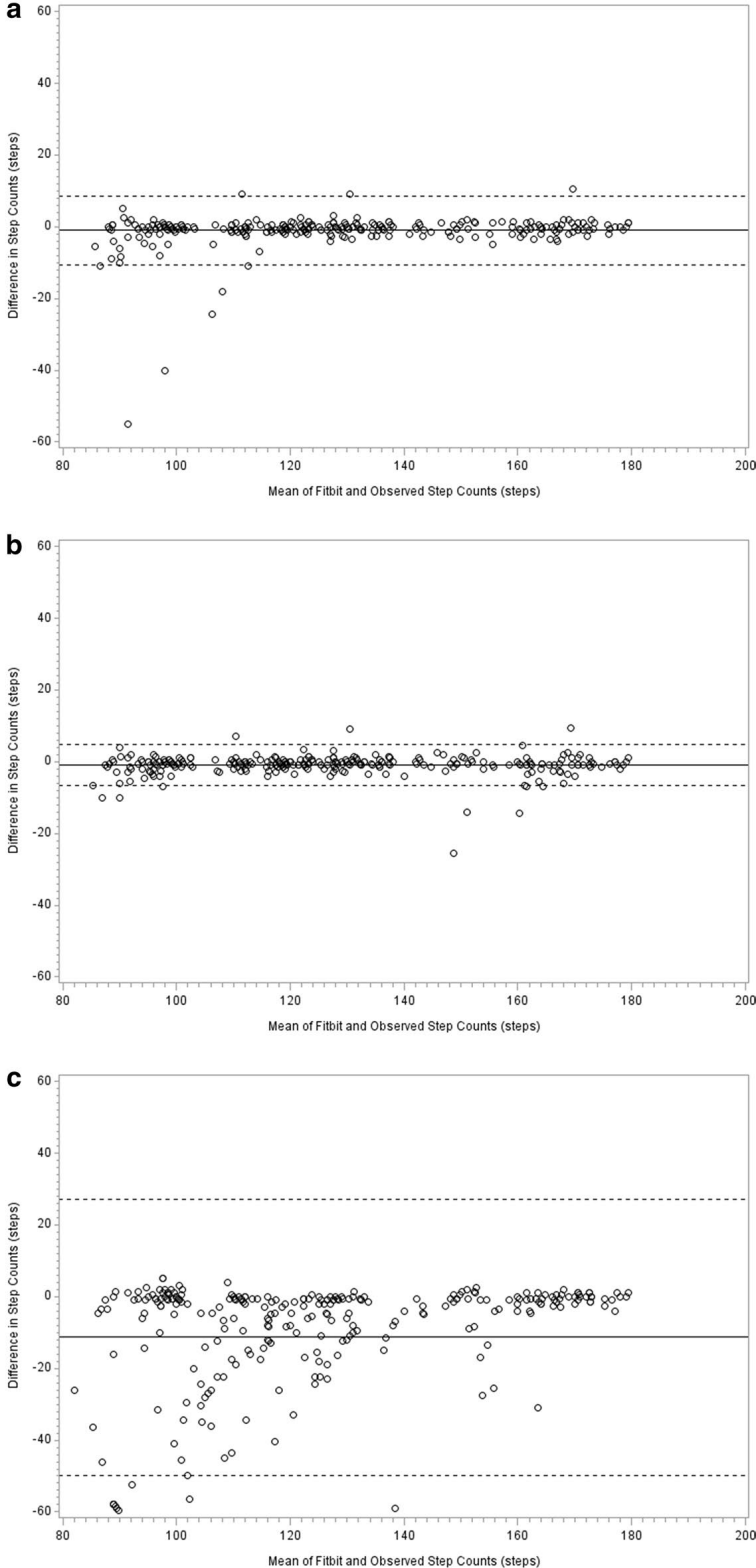
Bland Altman plots representing comparison between observed step counts (criterion) and Fitbit-estimated step counts for the upper torso-attached Fitbit One^®^ (**a**), hip-attached Fitbit One^®^ (**b**) and wrist-attached Fitbit Flex^®^ (**c**). *Solid lines* indicate the mean difference between observed step counts and Fitbit-estimated step counts, and *dashed lines* indicate limits of agreement

Estimated step counts from the Fitbit^®^ trackers showed moderate to substantial agreement with observed step counts. Across phases, the concordance correlation coefficients for the upper torso attachment site of the Fitbit One^®^, the hip attachment site of the Fitbit One^®^ and Fitbit Flex^®^ (each compared to observed step counts) were 0.98 (95 % CI 0.97–0.99), 0.99 (95 % CI 0.99–0.99), and 0.75 (95 % CI 0.70–0.79), respectively. Bland and Altman plots showed no apparent systematic bias for the upper torso attachment site of the Fitbit One^®^ and most points fell within the 95 % limits of agreement (Fig. 1). The limits of agreement for the upper torso attachment site (−10.8 to 8.7 steps) were wider than the hip attachment site of the Fitbit One^®^ (−6.5 to 4.7 steps), but markedly narrower than the Fitbit Flex^®^ (−49.9 to 27.1 steps). The observed and Fitbit-estimated step counts and relative percent errors are presented in Table 1 (upper panel). For the upper torso attachment site, the greatest difference was seen during slow walking as step counts were overestimated, on average, by 3.1 %; slightly exceeding the criterion validity threshold of 3.0 %. The relative percent errors were less than 1 % for all other phases. Step count estimates from the upper torso attachment site of the Fitbit One^®^ showed similar accuracy to that of the hip attachment site (0.1–3.1 % error vs. 0.2–1.5 % error) and were more accurate than the Fitbit Flex^®^ (2.1–15.8 % error). Comparison of step counts from the upper torso attachment site of the Fitbit One^®^ to both the hip attachment site and Fitbit Flex^®^ showed no significant differences between the upper torso and hip across all phases, but significantly higher step counts from the upper torso compared to the Fitbit Flex^®^ were observed during slow, moderate, and brisk walking.

**Table 1:** Criterion and Fitbit-estimated step counts (upper panel) and energy expenditure (lower panel) across treadmill speeds

Exercise phase	Observed step counts (steps)^a^	Fitbit One^®^ upper torso		Fitbit One^®^ hip		Fitbit Flex^®^ wrist	
		Estimated step counts	Percent error	Estimated step counts	Percent error	Estimated step counts	Percent error
Slow walk (1.9 mph)	98.2 ± 7.4	94.4 ± 6.8	−3.1 ± 7.8	96.2 ± 7.6*	−1.5 ± 2.8	83.2 ± 25.1*^‡^	−15.8 ± 27.9
Moderate walk (3.0 mph)	117.7 ± 6.1	117.0 ± 6.4	−0.6 ± 1.3	116.9 ± 6.4	−0.6 ± 1.2	106.2 ± 14.9*^‡^	−10.2 ± 12.3
Brisk walk (4.0 mph)	132.8 ± 7.4	132.1 ± 6.8	−0.2 ± 1.3	132.1 ± 7.0	−0.2 ± 1.4	117.4 ± 20.1*^‡^	−11.8 ± 14.7
Running (5.2 mph)	164.6 ± 8.9	164.2 ± 9.3	−0.1 ± 1.2	163.0 ± 9.9	−0.9 ± 2.9	161.2 ± 12.4	−2.1 ± 5.7

Exercise phase	Measured energy expenditure (kcal)^b^	Fitbit One^®^ upper torso		Fitbit One^®^ hip		Fitbit Flex^®^ wrist	
		Estimated energy expenditure (kcal)	Percent error	Estimated energy expenditure (kcal)	Percent error	Estimated energy expenditure (kcal)	Percent error
Slow walk (1.9 mph)	2.8 ± 0.5	2.5 ± 0.4*	−9.7 ± 14.9	2.6 ± 0.4*	−7.8 ± 15.2	5.2 ± 1.4*^‡^	83.4 ± 45.2
Moderate walk (3.0 mph)	3.8 ± 0.8	4.4 ± 1.0*	17.7 ± 37.2	4.1 ± 1.1	12.9 ± 43.3	6.4 ± 0.8*^‡^	68.3 ± 29.9
Brisk walk (4.0 mph)	5.6 ± 1.4	6.4 ± 1.2*	19.9 ± 44.2	6.0 ± 1.2^‡^	12.1 ± 45.0	7.2 ± 1.4*^‡^	29.4 ± 33.2
Running (5.2 mph)	8.3 ± 2.6	9.5 ± 2.4	10.8 ± 35.3	7.9 ± 1.2^‡^	−3.4 ± 32.8	10.6 ± 1.3*^‡^	24.5 ± 28.0

Data presented as mean ± standard deviation

* Significantly different from criterion at P ≤ 0.001

^‡^Significantly different from Upper Torso attachment site at P ≤ 0.001

^a^Manually counted by two observers from a video recording and averaged; 1-min epoch

^b^Measured by gas exchange indirect calorimetry; 1-min epoch

#### Energy expenditure

**Fig. 2:**
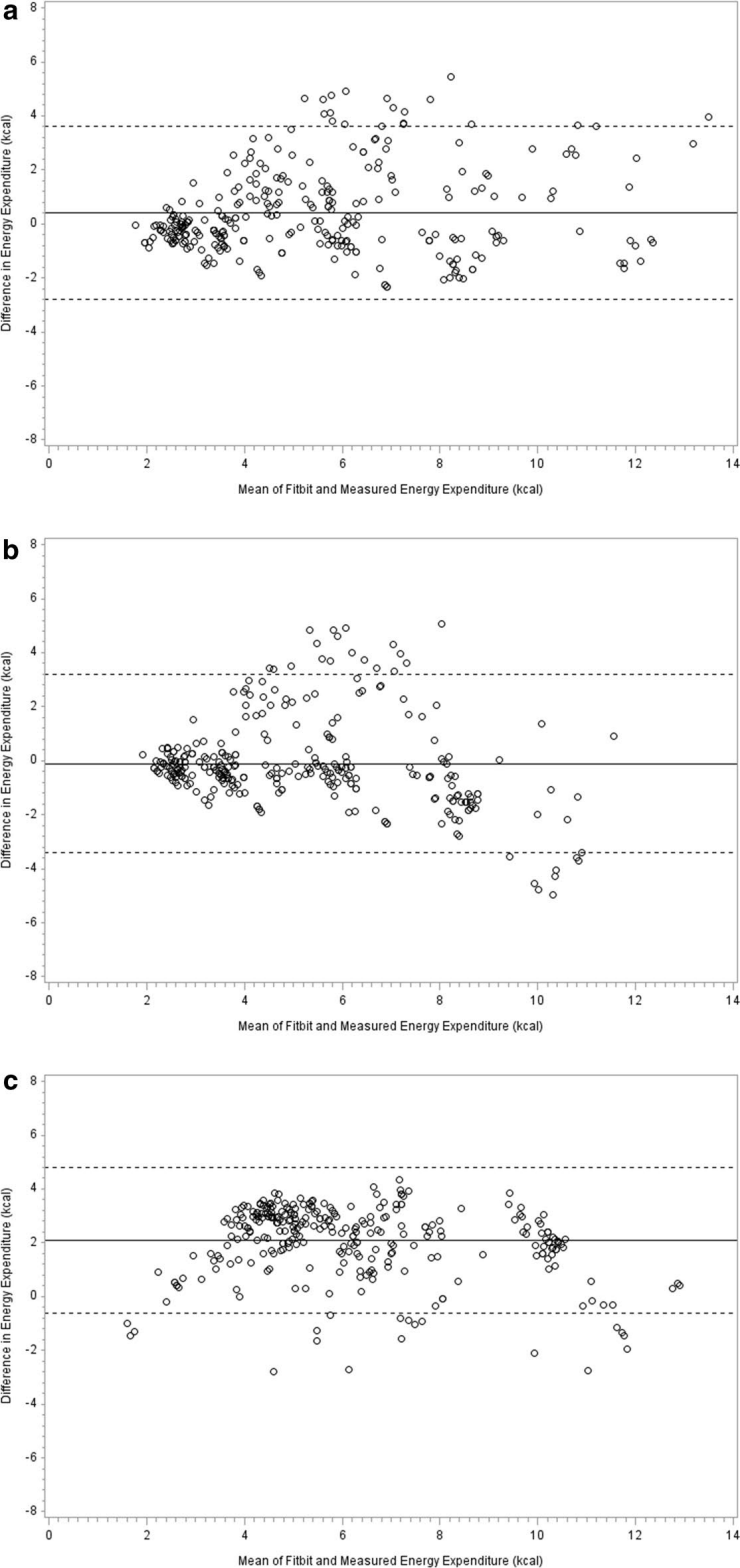
Bland Altman plots representing comparison between measured energy expenditure counts (criterion) and Fitbit-estimated energy expenditure for the upper torso-attached Fitbit One^®^ (**a**), hip-attached Fitbit One^®^ (**b**) and wrist-attached Fitbit Flex^®^ (**c**). *Solid lines* indicate the mean difference between measured energy expenditure and Fitbit-estimated energy expenditure, and *dashed lines* indicate limits of agreement

Estimated energy expenditure from the Fitbit^®^ trackers showed moderate agreement with energy expenditure measured by gas exchange indirect calorimetry. Across phases, the concordance correlation coefficients for the upper torso attachment site of the Fitbit One^®^, the hip attachment site of the Fitbit One^®^, and Fitbit Flex^®^ (each compared to energy expenditure measured by gas exchange indirect calorimetry were 0.82 (95 % CI 0.78–0.85), 0.77 (95 % CI 0.72–0.81), and 0.62 (95 % CI 0.57–0.67), respectively. Bland and Altman plots showed no apparent systematic bias for the upper torso attachment site of the Fitbit One^®^ and most points fell within the 95 % limits of agreement (Fig. 2). The limits of agreement width were similar across the upper torso attachment site of the Fitbit One^®^ (−2.8 to 3.6 kcal), hip attachment site of the Fitbit One^®^ (−3.4 to 3.2 kcal), and Fitbit Flex^®^ (−0.6 to 4.8 kcal). The indirect calorimetry-measured and Fitbit-estimated energy expenditure and relative percent error are presented in Table 1 (lower panel). For the upper torso attachment site, the percent error of Fitbit-estimated energy expenditure relative to the criterion ranged from −9.7 to 19.9 %; exceeding the criterion validity threshold of 3.0 % across all phases. The greatest differences were seen during moderate and brisk walking as energy expenditure was underestimated, on average, by 17.7 and 19.9 % during these stages, respectively. Energy expenditure estimates from the upper torso attachment site of the Fitbit One^®^ were, on average, less accurate than the hip attachment site (3.4–12.9 % error), but more accurate than the Fitbit Flex^®^ (24.5–83.4 % error). Comparison of estimated energy expenditure from the upper torso attachment site of the Fitbit One^®^ to both the hip attachment site and Fitbit Flex^®^ showed significant differences between the upper torso and hip during brisk walking and running, and between the upper torso and Fitbit Flex^®^ across all phases.

### Discussion

Our study shows that the upper torso attachment site of the Fitbit One^®^ yielded step count estimates during treadmill walking and running with relative percent errors of 3.1 % or less for all tested walking and running speeds. With relative percent errors of less than 1 % (less than a 1 step difference), the Fitbit One^®^ when attached to the upper torso was especially accurate at moderately paced and faster walking/running speeds. Energy expenditure estimates for the upper torso attachment site of the Fitbit One^®^ yielded relative percent errors that ranged from 9 to 19 %. These findings highlight that energy expenditure estimates from the Fitbit One^®^ attached to the upper torso provide a gross indication of engagement in physical activity; however, the 9–19 % error per minute could amount to large differences in total energy expenditure during a 24-h period.

The findings from the current study concur with other studies which have similarly found the Fitbit One^®^ to be highly accurate at measuring step counts [2, 14, 15]. Takacs et al. reported that the percent relative error was less than 1.3 % across five different treadmill walking speeds (0.9, 1.12, 1.33, 1.54, 1.78 m/s) relative to observed step counts for hip attachment of the Fitbit One^®^ in a population of 30 healthy adults [14]. More recently, in 500-step and 1500-step trials of treadmill walking at 3.0 mph, Case et al. reported the estimated mean step counts from hip attachment of the Fitbit One^®^ to be 498.6 ± 3.7 steps (0.3 ± 0.7 % error) and 1497.0 ± 10.7 steps (0.2 ± 0.7 % error), respectively, in a population of 14 healthy adults [2]. Our findings extend this previous research by indicating that the Fitbit One^®^ can accurately measure steps when worn at the upper torso attachment site.

Our finding that energy expenditure estimates for the upper torso attachment site of the Fitbit One^®^ yielded errors in the range of 9–19 % is also consistent with previous studies examining the validity of energy expenditure estimates from consumer-grade and research-grade accelerometers. Noah et al. found that the Fitbit Ultra^®^ (an earlier model of the Fitbit One^®^) at the hip attachment site yielded error of 10–12 % during treadmill walking (3.5 mph) and running (5.5 mph) relative to measured energy expenditure from indirect calorimetry among healthy young adults [16]. Error in the accuracy of energy expenditure estimates is common even among research-grade accelerometers. A systematic review by Van Remoortel et al. showed that among laboratory validation studies of the Actigraph (Models 7164/GT1 M), the most commonly tested accelerometer, the percent relative error ranged from −60.4 to −11.0, −25.8 to 25.8, −45.9 to 24.6, and −5.0 to 18.3 % during slow walking, intermediate walking, fast walking, and running, respectively [17]. As the Fitbit^®^ trackers (and all accelerometers) are movement sensors that assess a biomechanical aspect of physical activity, it would be unrealistic to expect perfect precision at estimating a physiological measure such as energy expenditure, particularly as individuals expend different levels of energy to achieve the same movements. Although a more precise measure may be required for assessing changes in energy expenditure over time, ascertainment of an individual’s general energy expenditure (for which only a strong correlation, not absolute accuracy, with true energy expenditure is needed) can still be attained with some certainty [17]. The correlation between estimated energy expenditure and measured energy expenditure at the upper torso attachment site (concordance correlation coefficient = 0.82) therefore suggests the Fitbit One^®^ may still have some utility as a tool to assess energy expenditure. Nevertheless, refinement of the Fitbit^®^ manufacturer’s prediction algorithm may be needed to provide a more accurate estimate of energy expenditure.

Relative to conventional attachment sites, the upper torso attachment site of the Fitbit One^®^ performed similarly to that of the hip attachment site for estimating steps and was better than the wrist-based model (Fitbit Flex^®^). For energy expenditure, the upper torso attachment site of the Fitbit One^®^ was less accurate than the hip attachment site, but more accurate than the Fitbit Flex^®^; although each tracker/attachment site yielded percent errors greater than 3 % for all tested walking and running speeds. These findings suggest the upper torso attachment site may be a viable alternative to the hip attachment site, albeit with some degree of decreased accuracy.

Previous studies have reported gender differences in the use and adoption of physical activity trackers; with female participants reporting concerns with the physical design of the trackers including device aesthetics (device being bulky and unattractive), wearability (not easy to conceal), and practical inconveniences (wrist jewelry preventing use of smart watch; dress with no pockets for clip-on device) [18, 19]. As the long-term use of commercial physical activity trackers has been reported to be poor (one-third stop using within six months) [20], strategies to overcome barriers to wear in female populations may be important to the success of future studies. By examining the accuracy of the upper torso attachment site of the Fitbit One^®^, our findings support its wear at an alternative attachment site that may permit better concealment than hip or wrist attachment and provide a clip-on attachment site more conducive for wear among females (e.g. dress wear). Furthermore, as there were no significant differences between step counts from the upper torso and hip attachment sites, our findings suggest that interchanging attachment sites (for example, changing attachment site on a given day depending on pants vs. dress wear; or permitting different attachment sites for males and females) is feasible in future studies that measure step counts as the primary exposure/outcome. A recent randomized controlled physical activity intervention reported that females were more favorable towards a clip-on Fitbit^®^ model (56 % preferred) than the wrist-based Fitbit^®^ model (20 % preferred; 24 % had no preference) [21]. Future studies, however, are still needed to determine if the upper torso attachment site would yield better participant acceptability and compliance than the hip attachment site.

Several limitations should be noted when interpreting our findings. First, our sample size is small. Nonetheless, it was sufficient to clearly depict measurement differences between the Fitbit^®^ trackers and criterion measures. Second, our study sample was comprised of healthy volunteers who were young-to-middle aged adults and normal-to-overweight. Differences in gait mechanics, speed of movement, and body composition have been reported to affect the raw acceleration signal and its conversion into step counts and energy expenditure [22, 23]. Thus, our findings may not be generalizable to older adult, obese, or musculoskeletal/neuromuscular impaired populations. Third, although the manufacturer specifies that the Fitbit One^®^ and Fitbit Flex^®^ both contain microelectromechanical triaxial accelerometers, the filtering and processing of acceleration signals for each tracker are not publically available. Thus, it is unclear whether the discrepancy in accuracy between the Fitbit One^®^ and Fitbit Flex^®^ was due to the attachment site or the tracker studied. Finally, testing was conducted on a treadmill to create a controlled environment to assess the accuracy of the Fitbit^®^ trackers. Relative to overground walking, treadmill walking has been associated with a shorter stride length and increased step cadence and width [24]. Future studies, therefore, may be needed to confirm the accuracy of upper torso attachment of the Fitbit One^®^ during overground walking. As the performance of accelerometers in a controlled laboratory setting may not translate fully to free-living conditions [25], future studies are also needed to assess the validity of the upper torso attachment site of the Fitbit One^®^ under free-living conditions.

In conclusion, our study shows that step counts obtained by the Fitbit One^®^ from the upper torso attachment site are generally accurate across different walking and running speeds in healthy female adults. With relative percent errors of 9–19 %, some caution, however, may be warranted when interpreting absolute energy expenditure estimates. The upper torso attachment site outperformed the wrist-based Fitbit Flex^®^ and yielded similar step count and energy expenditure estimates to that of hip attachment of the Fitbit One^®^. These data support the upper torso as an alternative attachment site for the Fitbit One^®^ among female adults.

## Abbreviations

NHANES: National Health and Nutrition Examination Survey
BMI: body mass index
